# Histone Carbonylation Is a Redox-Regulated Epigenomic Mark That Accumulates with Obesity and Aging

**DOI:** 10.3390/antiox9121210

**Published:** 2020-12-01

**Authors:** Amy K. Hauck, Tong Zhou, Ambuj Upadhyay, Yuxiang Sun, Michael B. O’Connor, Yue Chen, David A. Bernlohr

**Affiliations:** 1 Department of Biochemistry, Molecular Biology and Biophysics, University of Minnesota, Minneapolis, MN 55455, USA; Amy.Hauck@pennmedicine.upenn.edu (A.K.H.); tongzhou.simm@yahoo.com (T.Z.); yuechen@umn.edu (Y.C.); 2 Department of Molecular Biology, Cell Biology, Developmental Biology and Genetics, University of Minnesota, Minneapolis, MN 55455, USA; upad0023@umn.edu (A.U.); moconnor@umn.edu (M.B.O.); 3 Department of Nutrition, Texas A&M University, College Station, TX 77843, USA; yuxiangs@tamu.edu

**Keywords:** histone, carbonylation, adipose, epigenomics, 4-HNE (4-hydroxynonenal), 4-HHE (4-hydroxy hexenal), aging

## Abstract

Oxidative stress is a hallmark of metabolic disease, though the mechanisms that define this link are not fully understood. Irreversible modification of proteins by reactive lipid aldehydes (protein carbonylation) is a major consequence of oxidative stress in adipose tissue and the substrates and specificity of this modification are largely unexplored. Here we show that histones are avidly modified by 4-hydroxynonenal (4-HNE) in vitro and in vivo. Carbonylation of histones by 4-HNE increased with age in male flies and visceral fat depots of mice and was potentiated in genetic (*ob*/*ob*) and high-fat feeding models of obesity. Proteomic evaluation of in vitro 4-HNE- modified histones led to the identification of both Michael and Schiff base adducts. In contrast, mapping of sites in vivo from obese mice exclusively revealed Michael adducts. In total, we identified 11 sites of 4-hydroxy hexenal (4-HHE) and 10 sites of 4-HNE histone modification in visceral adipose tissue. In summary, these results characterize adipose histone carbonylation as a redox-linked epigenomic mark associated with metabolic disease and aging.

## 1. Introduction

Obesity is a chronic, low-grade inflammatory state in which increased pro-inflammatory signaling leads to oxidative stress, altered cellular metabolism, and the development of insulin resistance [1]. In response to high-fat feeding, resident immune cells and adipocytes produce pro-inflammatory cytokines and adipokines that potentiate inflammation, particularly through the accumulation and polarization of macrophages [1]. In the adipocyte, this inflammation is coincident with elevated oxidative stress, and dysregulation at the transcriptional level of major metabolic pathways in response to oxidative stress is a phenotypic hallmark of obesity [1,2]. In particular, obesity-induced inflammation is associated with the downregulation of several genes associated with mitochondrial antioxidant biology such as glutathione S-transferase (Gsta4), aldehyde dehydrogenase (Aldh1), peroxiredoxin 3 (Prdx3) and glutathione peroxidase (Gpx4), as well as enzymes of the branched chain amino acid and tricarboxylic acid pathways [3,4,5]. In murine systems, such changes occur exclusively in the visceral depot and spare the subcutaneous depot whereas in humans both depots undergo metabolic reprogramming as a function of inflammation. Consistent with adipose genes linked to mitochondrial biology being key regulatory targets, clinical treatment of insulin resistance with thiazolidinediones results in the upregulation of adipocyte genes targeting mitochondrial pathways [6,7].

Paralleling evidence reveals the crosstalk between inflammation and adipose mitochondrial biology, and recent work from Schaum et al. [8] on gene expression and Yu et al. [9] on targeted pathway proteomics have both indicated that adipose tissue may serve as a sentinel of age-dependent processes. Both reports indicate that mitochondria from white adipose are reprogrammed as a function of age and adiposity, highlighting the importance of oxidative stress response pathways in metabolic disease. Interestingly, Yu et al. report that the protein expression levels of Gsta4, Prdx3 and Aldh2 are all downregulated as a function of age in lean adipose tissue [9]. 

Despite these observations, the precise mechanisms that link inflammation and aging in adipose metabolic dysregulation remain enigmatic. Oxidative protein damage represents a potential mechanistic target for metabolic control since it is directly correlated with the expression of antioxidant genes and levels of reactive oxygen species [4,10]. In the obese state, adipose oxidative stress is linked to increased levels of all major ROS species including superoxide anion, hydrogen peroxide and hydroxyl radicals [8,9]. Hydroxyl radicals attack the acyl chains of polyunsaturated lipids (either free fatty acids, triglycerides or membrane phospholipids) ultimately leading to the formation of highly reactive, diffusible lipid aldehydes. These aldehydes, such as 4-hydroxynonenal (4-HNE) and 4-hydroxyhexenal (4-HHE), are subject to nucleophilic attack by the side chains of lysine, cysteine, and histidine residues, resulting in covalent lipid adducts [11]. Although the modification is chemical, it is clear that specific proteins are modified [10,11,12,13,14,15]. In addition, a recent report suggests that de-carbonylation may be an enzymatically-driven process and that the steady state level of carbonylation is driven by the balance between chemical modification and enzymatic de-modification [12]. 

In the obese state, protein carbonylation in adipose tissue is increased three-fold, leading to protein dysfunction and altered mitochondrial output [13,14]. Surprisingly, recent work from Hauck et al. has shown that the nucleus is a major site for protein carbonylation in adipose tissue [15]. This was an unanticipated result that suggests a new mechanistic connection between oxidative stress and nuclear regulatory events. Indeed, proteomic evaluation of nuclear carbonylation targets revealed that many transcriptional and chromatin-associated regulatory proteins are modified in the obese state [15]. 

Herein, we expand upon prior studies and describe the post-translational modification of histones by reactive lipid aldehydes 4-HNE and 4-HHE. Histone modifications are widely associated with either transcriptional repression or activation via acetylation and methylation, [16,17], while the effects of other modifications are much more varied depending on the substrate (phosphorylation, sumoylation, etc.). We find that the core histones are specific targets of carbonylation and that these modifications accumulate in adipose tissue as a consequence of high-fat feeding (HFD) and of aging. Furthermore, 4-HNE and 4-HHE, despite their chemical similarity, modify different sites, introducing the concept that each oxidation event may initiate distinct signaling outcomes. 

## 2. Materials and Methods 

### 2.1. Animals 

Wild-type C57Bl/6J mice were weaned at 3 weeks of age and fed either a high-fat diet (20% protein by weight, 35.5% fat by weight (60% by calories), 36.3% carbohydrate by weight; BioServ F3282) or a chow diet for 12 weeks. *Ob*/*ob* mice were obtained at 10 weeks of age and maintained on chow diet for two weeks prior to sacrifice. Animals were sacrificed by cervical dislocation and epididymal and inguinal white adipose depots were removed for analysis. All experiments with mice were approved by the University of Minnesota Institutional Animal Care and Use Committee under protocol number 1501-32208A or the Texas A&M Institutional Animal Care and Use Committee under protocol number 2019-0378.

### 2.2. Drosophila Melanogaster Flies (yw) 

Drosophila melanogaster flies were reared on standard agar-cornmeal medium at 25 °C under a 12:12 h light/dark cycle. Male and virgin female flies were collected within 14 h of eclosion and housed separately (50–60 flies per vial). Flies were counted every week and transferred to new vials every two days for the duration of the experiment. At the indicated time points, flies were collected, snap frozen in liquid nitrogen, and stored at −80 °C until processing.

### 2.3. Cell Culture 

The 3T3-L1 fibroblasts were grown to confluence and differentiated as previously described [18] using the standard differentiation cocktail consisting of dexamethasone, methylisobutylxanthine, and insulin. On day 7 of differentiation, cells were treated with 0.5 nM TNFα or vehicle in DMEM +5% FBS for 24 h before harvesting.

### 2.4. Histone Preparation

From mice, fresh tissue was minced on ice in hypotonic lysis buffer (10 mM Tris-Cl pH 8.0, 1 mM KCl, 1.5 mM MgCl_2_, 1mM DTT) with protease and phosphatase inhibitors. Tissue was homogenized with a glass-teflon system using an electric homogenizer (8 strokes, 1600 rpm), centrifuged at 1000 rpm at 4 °C for 10 min and the fat cake discarded. Samples were briefly vortexed and centrifuged at 3700 rpm at 4 °C for 10 min to collect nuclei. Histones were purified using acid extraction following the method described by Schecter et al. [19], reduced in 15 mM NaBH_4,_ and quantitated using the bicinchoninic acid assay (Sigma Aldrich, St Louis, MO, USA). From flies, animals were ground using mortar/pestle and processed as described above.

### 2.5. Immunoblotting Analysis 

Samples were resolved by SDS-PAGE, wet transferred to a nitrocellulose membrane, blocked for 1 h with blocking buffer (LI-COR 927-4000, Lincoln, NE, USA) and incubated with primary antibodies overnight at 1:1000 dilutions. Membranes were then washed and incubated for 1 h with fluorescently labeled secondary antibodies (LI-COR) and imaged using the LI-COR Odyssey Imager. Antibodies used include 4-HNE (Millipore 393207, Burlington, MA, USA), 4-HHE (a generous gift of Matthew Picklo, Human Nutrition Research Center, Grand Forks, ND), Histone H3 (abcam 10799, Cambridge, UK), and Histone H4 (Cell Signaling 2935S).

### 2.6. In Vitro Modification of Purified histones

Acid-extracted histones were resuspended in adduction buffer (100 mM potassium phosphate pH 7.4, 150 mM NaCl) at 37 °C with the indicated concentrations of 4-HNE (Cayman Chemical 32100, Ann Arbor, MI, USA) or 4-HHE (Cayman Chemical #32060). The reaction was quenched with the addition of 5 mM dithiothreitol and modification was verified by immunoblotting.

### 2.7. Immunoprecipitation of Carbonylated Proteins

For immunoprecipitation of carbonylated proteins, 10–20 ug of reduced, purified histones was diluted to 500 uL with a phosphate-buffered saline containing 1% NP40 and then pre-cleared with protein A/G agarose resin (Santa Cruz sc-2003). 4-HNE primary antibody (Millipore 393207) crosslinked to protein A/G agarose resin using DMP imidoester crosslinking reagent (Thermo Scientific) was incubated with histones overnight. Following complex formation, the resin was washed three times with IP buffer (50 mM Tris-HCl pH 7.5, 150 mM NaCl, 1% Triton X-100, 0.5% SDS) and proteins were eluted by boiling in sample loading buffer. Samples were resolved by SDS-PAGE and stained with Imperial Protein Stain (Thermo Scientific) prior to in-gel digestion. 

### 2.8. In-Gel Digestion

SDS-PAGE gel bands corresponding to histone H1, histone H2A/B and 3, or histone H4 were washed with 50% EtOH overnight and then with water for one hour. Each gel band was cut into small pieces and dehydrated using acetonitrile. The gel pieces were washed sequentially using 50 mM ammonia bicarbonate, 100% acetonitrile, and dried completely by SpeedVac (ThermoFisher, Waltham, MA, USA). Trypsin stock solution (Promega, Madison, WI, USA) was reconstituted in 50 mM ammonia bicarbonate buffer to 10 ng/µL and applied to digest proteins in gel overnight at 37 °C. Peptides were extracted sequentially using 5% trifluoroacetic acid and 50% acetonitrile in water (*v*/*v*) and 100% acetonitrile. The pooled peptide extracts were dried in SpeedVac and desalted by C18 Stagetip as previously described [20]. 

### 2.9. LC–MS/MS

Peptides were reconstituted in HPLC buffer A (0.1% formic acid in water, *v*/*v*) and injected into a Proxeon Easy nLC 1000 HPLC system (ThermoFisher) by autosampler. Peptides were separated by C18 column packed in-house (15 cm × 75 µm, ReproSil-Pur Basic C18, 2.5 µm, Dr. Maisch GmbH) with a linear gradient of 5–35% HPLC buffer B (0.1% formic acid in acetonitrile, *v*/*v*) at a flow rate of 200 nL/min. Eluted peptides were directly electrosprayed into the Fusion Orbitrap mass spectrometry (ThermoFisher). The instrument was operated in a data-dependent mode and full mass spectra were acquired with a resolution of 120,000 FWHM at 200 *m*/*z* and MS/MS spectra were acquired using collision-induced dissociation (CID) with 35% collision energy for detection in the ion trap.

### 2.10. MS Data Processing

Raw MS data were processed by MaxQuant software (v 1.5.3.12) for protein identification [21]. Variable modifications include acetylation on lysine, methylation on lysine or arginine, methionine oxidation, protein N-terminal acetylation, HNE and reduced HNE on cysteine, histidine and lysine, as well as HHE and reduced HHE on cysteine, histidine and lysine (excluding C-terminal lysine modification on peptides). Tryptic and semi-tryptic proteolytic cleavages were considered and a maximum of four missing cleavages were allowed. The precursor ion mass tolerance was set to +/−4.5 ppm and the fragment ion mass tolerance was set to +/−0.5 Da. MS data were searched against a custom NCBI mouse histone database concatenated with common contaminant and decoy protein sequences. Peptides were filtered with a 1% False Discovery Rate (FDR) at the peptide, protein and modification site levels. A minimum Andromeda score of 40 was required for the identification of modified peptides.

### 2.11. Statistical Methods

Data are presented as the mean ± SEM unless otherwise indicated. Graphpad Prism 7 software was used for analysis of Western blot quantification and for survival curve analysis, while Maxquant software was used for PTM identification. For survival curve comparison, we used the Log-rank (Mantel–Cox) test to calculate a P value. For Western blots, one-way ANOVA was used to compare between groups where *p* < 0.05 was considered to be statistically significant and P values are indicated by the markers * *p* < 0.05, ** *p* < 0.005, *** *p* < 0.0005, and **** *p* < 0.00001 when comparing to either 0 weeks (flies) or 3 months (mice); # *p* < 0.05, ## *p* < 0.005, ### *p* < 0.0005, #### *p* < 0.00001 when comparing to either 2 weeks (flies) or 6 months (mice). 

## 3. Results

### 3.1. The Core Histones Are Carbonylated In Vitro and In Vivo

Hauck et al. previously demonstrated that protein carbonylation is elevated in the nucleus of adipocytes from obese C57Bl/6J mice compared to lean controls [15]. Moreover, the increase in nuclear protein carbonylation was focused on the visceral fat depot, as the subcutaneous fat depot did not exhibit increased obesity-linked nuclear protein carbonylation. Consistent with this, Long et al. have shown that the mRNA expression of mitochondrial antioxidants glutathione S-transferase a4 (Gsta4), aldehyde dehydrogenase 2 (Aldh2), peroxiredoxin 3 (Prdx3) and glutathione peroxidase 4 (Gpx4) are each downregulated in visceral, but not subcutaneous depots from 15 week old obese mice [4]. Gsta4 and Aldh2 are two critical enzymes responsible for detoxification of reactive lipid aldehydes and downregulation of these enzymes results in increased levels of 4-hydroxynonenal (4-HNE) and 4-oxononenal (4-ONE) in the visceral depot of obese mice. 

**Figure 1:**
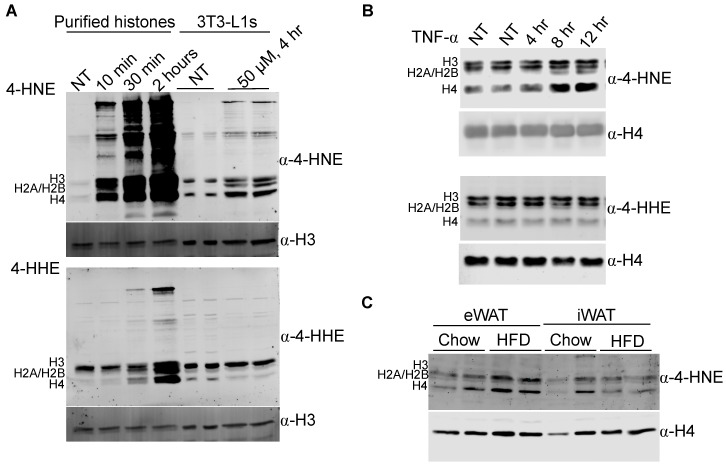
4-HNE modification of histones occurs in vivo and is increased in obese animals. (**A**) Western blot of in vitro modification of histones with 4-HNE (upper panel) and 4-HHE (lower panel). Acid-extracted histones from 3T3-L1 adipocytes were modified by 4-HNE or 4-HHE for the indicated time in vitro (lanes 1–4). 3T3-L1 adipocytes were treated on day 8 of differentiation with vehicle, 4-HNE (upper), or 4-HHE (lower), followed by histone extraction and analysis by Western blot (lanes 5–8). (**B**) 3T3-L1 adipocytes treated with 0.5 nM TNFα on day 8 of differentiation for 4, 8, and 12 h. 4-HNE (upper) and 4-HHE (lower) modifications on purified histones. (**C**) 4-HNE modification of histones purified from eWAT and iWAT of chow- and high-fat-diet-fed (HFD) mice. NT indicates no treatment (vehicle) control.

The core histones are relatively small, lysine-rich proteins (10–15 kDa) and are among the most abundant proteins in the nucleus. Analysis of nuclear protein carbonylation utilizing antibodies targeting 4-HNE adducts revealed a number of low molecular weight polypeptides [15] (Figure S1A), that suggested histones may be nuclear carbonylation substrates. To test this, we first asked whether histones could be modified by 4-HNE. Acid extraction of histones from 3T3-L1 adipocytes (Figure S1B. left) followed by in vitro incubation with 4-HNE demonstrated that histone-4-HNE adducts form readily in vitro on all four core histones (Figure 1A). Furthermore, we were able to detect basal 4-HNE modification of core histones from day 8 3T3-L1 adipocytes, and treatment of cells with 4-HNE resulted in increased modification (Figure 1A). Together, these results indicate that histones are readily modified by 4-HNE both in solution and in culture. In contrast, incubation of purified histones with 4-HHE or treatment of adipocytes with 4-HHE also resulted in histone-lipid adducts, but to a lesser degree than 4-HNE, suggesting that, despite their chemical similarity, 4-HNE and 4-HHE exhibit disparate kinetics in the formation of lipid adducts on histones (Figure 1A).

To assess whether histone carbonylation was dynamically regulated, we turned to oxidative stress models using cultured 3T3-L1 adipocytes. In 3T3-L1 adipocytes, treatment of cells with the inflammatory cytokine TNFα is known to down regulate the expression of mitochondrial antioxidant Gsta4 [22] and increase protein carbonylation [3,22]. Cells treated with TNFα exhibited increased H2A/H2B, H3 and H4 modification with 4-HNE but not with 4-HHE (Figure 1B). Extending this finding, silencing of glutathione-S-transferase A4 (Gsta4) in 3T3-L1 adipocytes resulted in elevated 4-HNE modification of histones (Figure S1D).

We next assessed histone carbonylation in vivo. Acid extraction of histones from epididymal white adipose tissue (eWAT) and inguinal white adipose tissue (iWAT) depots of lean and obese mice followed by Western blot analysis of 4-HNE adducts demonstrated a significant increase in 4-HNE adducts on all core histones from eWAT depots from mice fed a high-fat diet for 12 weeks (HFD) compared to lean controls (Figure S1B, Figure 1C). To rule out diet-specific effects, we performed the same analysis with the histone fraction from adipose tissue of lean wild-type (WT) and *ob*/*ob* mice (Figure S1C). *ob*/*ob* mice exhibited a similar increase in histone carbonylation in eWAT but not in iWAT, indicating that histone carbonylation is not linked to the diet composition per se, but is the result of increased adiposity and depot (Figure S1C). Together, these data indicate that histone carbonylation is a dynamic modification that occurs in adipocytes both in vitro and in vivo.

### 3.2. Histone Carbonylation Accumulates in Aged Flies and Mice

**Figure 2:**
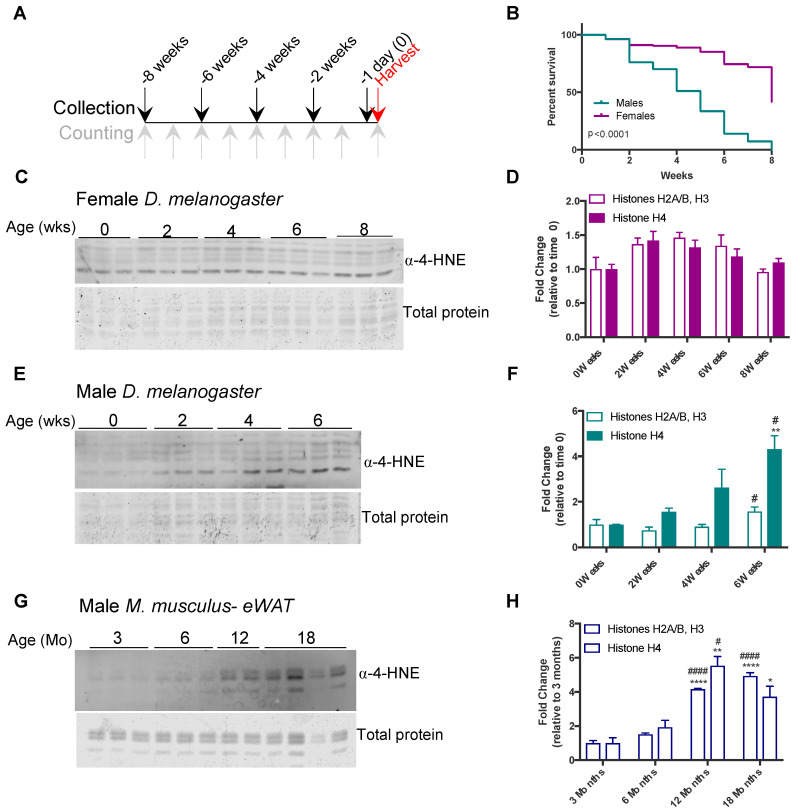
Histone carbonylation increases with age in mice and flies. (**A**) Schematic of *D. melanogaster* aging experimental design. Flies were collected every two weeks for 8 weeks and aged until the end of the study (harvest). Flies were counted and dead animals removed each week. (**B**) Survival curve of flies over the course of 8 weeks. *p*-Value determined by Log-rank test. (**C**,**E**) 4-HNE modification of histones purified from total fly homogenate for female and male flies, respectively. (**D**,**F**) Quantitation of Western blots from (**C**,**E**) normalized to total protein, relative to 0 weeks. (**G**) 4-HNE modification of histones purified from eWAT of mice aged 3–18 months. (**H**) Quantitation of Western blot from (**G**) normalized to total protein, relative to 3 months). In (**D**,**F**,**H**) results were compared by one-way ANOVA. * indicates significance compared to time 0 (0 weeks or 3 months). # indicates significance relative to 2 weeks in flies and 6 months in mice. (* *p* < 0.05, ** *p* < 0.005, **** *p* < 0.00001, #### *p* < 0.00001, # *p* < 0.05).

The observation that histone carbonylation is induced in 3T3-L1 adipocytes in response to inflammatory stimuli suggests that histone carbonylation is redox regulated. To test other models in which oxidative stress plays a defining role independent of obesity we turned our attention to models of aging. Aging is a complex process that involves many different metabolic perturbations, including elevated ROS levels, and increased oxidative damage to biomolecules is one of the pillars of aging [23]. Several studies demonstrate the age-dependent increase in ROS and ROS-related damage, including modification of proteins by lipid electrophiles [24,25,26]. To test whether aging led to an accumulation of 4-HNE-modified histones, we aged virgin male and female Drosophila melanogaster flies for 8 weeks and assessed the viability and level of 4-HNE modification of histones from whole animals at 0, 2, 4, 6, and 8 weeks (Figure 2A). Consistent with previous aging studies [27], female flies exhibited a significantly higher survival rate than the males (Figure 2B). In females, we did not detect significant changes in histone carbonylation throughout the time course (Figure 2C,D). In contrast, male flies showed a trend towards increased modification of histone H4 by four weeks of age and a significant increase in 4-HNE modification of all core histones by 6 weeks of age (Figure 2E,F).

To parallel the age-dependent change in histone carbonylation in flies with a mammalian system, male mice were fed a chow diet and adipose histone carbonylation was evaluated at 3, 6, 12, or 18 months of age. As shown in Figure 2G,H, there was a significant increase in the level of histone modification of all core histones beginning at 12 months of age. Together, these data provide evidence that 4-HNE modification of histones is an evolutionarily conserved process that increases as a consequence of age.

### 3.3. Proteomic Analysis of In Vitro Histone Carbonylation Sites

**Figure 3:**
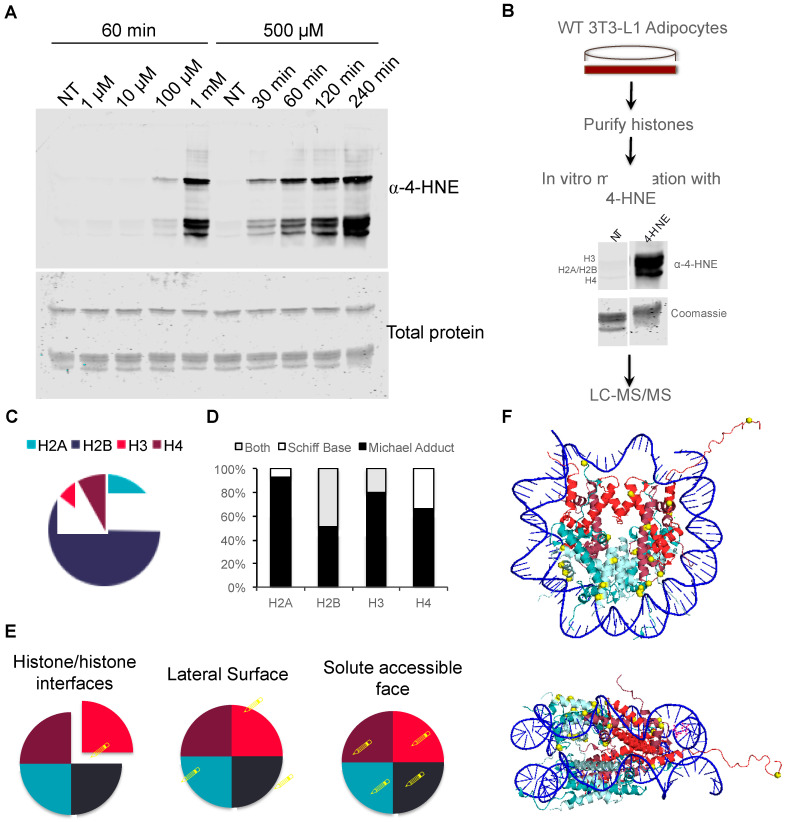
Proteomic evaluation of in vitro 4-HNE-modified core histones. (**A**) In vitro modification of purified histones with 4-HNE. Titration of 4-HNE concentration (left) and reaction time (right). (**B**) Flow diagram of LC–MS/MS workflow for proteomic analysis of modified sites. (**C**) Pie chart of distribution of 4-HNE modification across the core histones (**D**) Ratio of Michael adducts and Schiff bases identified on in vitro-modified histones. (**E**) Depiction of the categories of localization of identified sites on the nucleosome structure. (**F**) Ribbon diagram of the nucleosome (structure generated from pdb1aoi). 4-HNE-modified residues are shown in yellow.

To identify specific sites of histone modification by 4-HNE, we carried out proteomic analysis on histones modified both in vitro and in vivo. First, we assessed sites of modification in vitro in order to establish a proteomic workflow (Figure 3A,B). We incubated histones purified from 3T3-L1 cells with 4-HNE and analyzed the sites of modification as a function of time and concentration to establish standard conditions (Figure 3A). In vitro modification led to the robust modification of all core histones at the same rate, indicating that there is not a detectable site preference for 4-HNE modification in vitro (Figure 3A). 4-HNE modification of proteins most commonly occurs as either a Michael adduct on side chains of lysine, histidines, and cysteines or as a Schiff base on lysine residues [11]. Proteomic evaluation of in vitro-modified histones revealed multiple types of adducts on all core histones, but the majority of identified sites were Michael adducts (Figure 3C,D). These results are consistent with previous work indicating that Schiff base modification is labile and reversible, while Michael adducts are stable and non-reversible [11].

The core histone proteins share in common the basic structure of a C-terminal globular histone fold domain and an N-terminal, unstructured, lysine-rich tail. The lysine residues of the histone tail have been the subject of intense study due to their role in replication and epigenomic transcriptional regulation. Interestingly, over 90% of the in vitro-modified 4-HNE sites mapped to the globular histone-fold domain rather than the N-terminal lysine-rich tail (Table S1). Modifications were identified on all faces of the nucleosome, including residues involved in histone–histone interaction, nucleosome–DNA interaction on the lateral surface, and solute accessible surfaces (Figure 3E,F).

### 3.4. Proteomic Analysis of Histone Carbonylation Sites In Vivo

**Figure 4:**
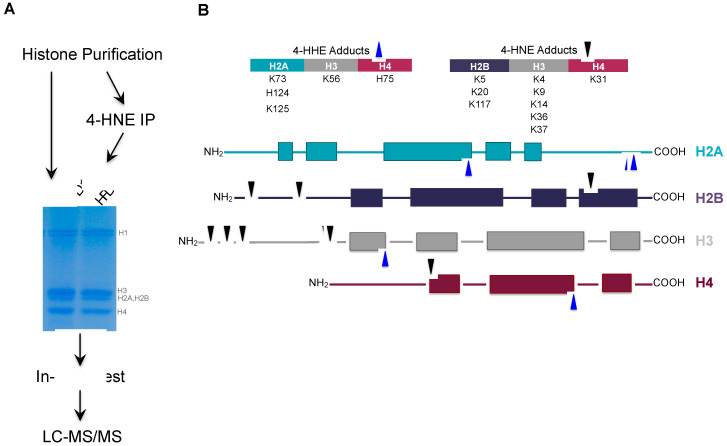
Identification of carbonylation sites in vivo. (**A**) Proteomic workflow for identification of sites. Both unenriched and immunoaffinity enrichment for 4-HNE-modified histones were resolved by SDS-PAGE, in gel digested, and analyzed by LC–MS/MS. (**B**) Cartoon diagram of the 4-HHE and 4-HNE adducts identified on core histones.

To compare the sites of histone carbonylation in vivo to those determined in vitro, we carried out carbonylation analysis of eWAT from HFD-fed animals using an unbiased approach in which total histones were purified from eWAT and resolved by SDS-PAGE prior to in gel-digestion and LC-MS/MS (Figure 4A). We also utilized an enrichment approach, whereby 4-HNE-modifed proteins were immunoprecipitated from the total histone pool prior to SDS-PAGE. Analysis of 4-HNE and 4-HHE adducts led to the identification of 21 histone modifications including 14 sites on the core histones and 7 sites on linker histone H1 (Figure 4B, Table S2). Of the sites identified, 20 are novel sites of carbonylation by lipid aldehydes. H2BK117 has previously been reported as a 4-oxononenal (4-ONE) target site in in vitro-modified cells [28]. Interestingly, of the 14 sites identified on core histones, 5 were exclusively modified with 4-HHE, while 9 were modified only with 4-HNE. We identified no carbonylation sites modified by more than one lipid aldehyde (Table S2). In contrast to in vitro-modified histones, we observed seven sites localized to the N-terminal histone tail including critical regulatory sites H3K4, H3K9, H3K36 and H2BK5.

Interestingly, the 4-HHE sites were all located within the C-terminal histone fold domain, while the 4-HNE modifications were localized primarily to the N-terminal histone tail (Figure 4B). Consistent with these data, analysis of histones from WT and *ob*/*ob* mice by Western blot indicates that 4-HNE modification is localized to H3 and H4, while 4-HHE is predominately found on H4 (Figure S1C). This is in contrast to purified histones modified in vitro, where all core histones display similar patterns of modification (Figure 1A). Together, these data suggest a previously unappreciated specificity for 4-HNE vs. 4-HHE modifications.

## 4. Discussion

The molecular and mechanistic linkage between environmental queues, metabolic disease and aging is complex and likely brought about by a combination of hormonal, metabolic and genetic determinants [29,30,31]. A common theme in many metabolic diseases is oxidative stress and downstream events linked to redox biology [32,33]. Here we examine the role of one such redox event, the covalent modification of proteins with reactive lipid aldehydes, as a major regulatory mechanism linking mitochondrial oxidative stress to the epigenome.

In this study, we show that the core histones are differentially modified by reactive lipid species 4-HNE and 4-HHE in obese white adipose depots. Furthermore, we show that these modifications are elevated in eWAT as a consequence of age. These effects are specific to epididymal depots, where previous work demonstrates a significant downregulation of major antioxidants in murine models of obesity, including the primary mammalian enzyme responsible for 4-HNE detoxification, Gsta4 [4]. In aging models, it is interesting to note that carbonylation was not elevated in female flies with age, while there was a significant increase in males. It has been well documented that female flies have a longer life expectancy than males [34]. This has been attributed to a variety of factors, but it has been demonstrated that female flies generally express higher levels of antioxidants and have less ROS production than males [27,35]. This may mitigate the deleterious effects of ROS and prevent modification of histones in females during the aging process. Importantly, work carried out in RKO cells treated with exogenous 4-HNE and 4-ONE also revealed that histone proteins are susceptible to carbonylation [28]. Our study expands upon these findings to demonstrate that histone modification occurs in vivo.

The epigenome is operationally defined by a complex and highly regulated series of biochemical modifications focused on the covalent modification of DNA or DNA binding proteins (largely histones) [36,37,38,39]. Such modifications lead to altered local chromatin architecture and the recruitment and assembly of transcription factors and DNA binding proteins, affecting either activation or repression of nearby genes. Indeed, while histone modification by methylation and acetylation are widespread and function in the control of chromatin organization and transcription, expanded analysis advanced via proteomic interrogation has revealed a series of additional modifications whose role(s) are less widely understood [36,39].

In this study, we identified 21 histone modifications including adducts on critical regulatory lysine residues H3K4, H3K9, H2BK5, and H3K56. The breadth of sites found in this study and their importance in transcriptional regulation underlie the functional importance of these findings. For example, H3K4me^3^ is a canonical activating mark [40] that is estimated to be present on up to 75% of human gene promoters [41,42]. In contrast, H3K9me^3^ is a strongly repressive mark when localized to promoter regions [43], while H3K9ac is associated with active transcription [44]. These modifications are dynamic and highly regulated by specific classes of enzymes that add or remove each post-translational modification. Importantly, the carbonylation of lysine residues via a Michael addition reaction is a covalent modification for which there are currently no known removing enzymes. This is critical for it implies that histone carbonylation is a stable epigenomic mark that persists under a variety of metabolic conditions until the histone protein itself is degraded. Moreover, since the reaction is non-reversible, it negates target sites from subsequent modification and further epigenomic control, potentially leading to prolonged impact on transcriptional regulation.

The differences in 4-HHE and 4-HNE modification is striking, both in Western blots and by proteomic evaluation of the sites modified. It is unclear why or how this selectivity occurs, particularly as carbonylation is a chemically driven process. These observations suggest that there is an unappreciated regulatory mechanism, either biological or chemical, that directs these modifications.

Currently, it is unclear whether histone carbonylation is targeted to specific sites in the genome. Generation of site-specific 4-HNE antibodies will enable studies aimed at determining genome-wide localization of 4-HNE PTM’s using chromatin immunoprecipitation-seq approaches. In addition, it will be important to examine whether histone carbonylation is a modification of opportunity in that it modifies any free histone lysine residue that the lipid encounters, resulting in genome-wide histone damage or whether it is more acutely regulated and accumulates in certain chromatin landscapes. Finally, it is unknown how the carbonylation of histones affects protein turnover. Future studies aimed at assessing turnover of modified histones will be informative in order to assess whether in metabolic disease and aging the elevated carbonylation is due to slow accumulation of adducts over time or rapid production of adducts that overcome slow histone turnover.

## Supplementary Materials

The following are available online at https://www.mdpi.com/2076-3921/9/12/1210/s1, Figure S1. (A) Western blot of 4-HNE adducts in crude nuclear extracts and post-nuclear extracts from eWAT of chow-fed and HFD-fed male mice. (B) Coomassie stain of acid-extracted histones separated by SDS-PAGE from 3T3-L1 adipocytes (left) and eWAT (right). (C) 4-HHE and 4-HNE modification of histones purified from eWAT (upper) and iWAT (lower) for WT and *ob*/*ob* mice. (D) 4-HNE adducts on histones purified from Scr control and Gsta4 knockdown (KD) 3T3-L1 adipocytes. Table S1: 4-HNE modifications identified on in vitro-modified histones.

## Abbreviations

4-HNE—*trans*-4-hydroxy-2-nonenal; 4-HHE—*trans*-4-hydroxy-2-hexenal; ROS—reactive oxygen species; eWAT—epididymal adipose tissue; iWAT—inguinal white adipose tissue; GSTA4—glutathione-S-transferase A4; HFD—high-fat diet; 4-ONE—4-trans-oxo-2-nonenal; PTM—post-translational modification.

## References

[1] Olefsky J.M., Glass C.K. (2010). Macrophages, Inflammation, and Insulin Resistance. Annu. Rev. Physiol..

[2] Furukawa S., Fujita T., Shimabukuro M., Iwaki M., Yamada Y., Nakajima Y., Nakayama O., Makishima M., Matsuda M., Shimomura I. (2004). Increased oxidative stress in obesity and its impact on metabolic syndrome. J. Clin. Investig..

[3] Hahn W.S., Kuzmicic J., Burrill J.S., Donoghue M.A., Foncea R., Jensen M.D., Lavandero S., Arriaga E.A., Bernlohr D.A. (2014). Proinflammatory cytokines differentially regulate adipocyte mitochondrial metabolism, oxidative stress, and dynamics. Am. J. Physiol. Endocrinol. Metab..

[4] Long E.K., Olson D.M., Bernlohr D.A. (2013). High-fat diet induces changes in adipose tissue trans-4-oxo-2-nonenal and trans-4-hydroxy-2-nonenal levels in a depot-specific manner. Free Radic. Biol. Med..

[5] Burrill J.S., Long E.K., Reilly B., Deng Y., Armitage I.M., Scherer P.E., Bernlohr D.A. (2015). Inflammation and ER Stress Regulate Branched-Chain Amino Acid Uptake and Metabolism in Adipocytes. Mol. Endocrinol..

[6] Bogacka I., Xie H., Bray G.A., Smith S.R. (2005). Pioglitazone Induces Mitochondrial Biogenesis in Human Subcutaneous Adipose Tissue In Vivo. Diabetes.

[7] Hondares E., Mora O., Yubero P., Rodriguez de la Concepción M., Iglesias R., Giralt M., Villarroya F. (2006). Thiazolidinediones and rexinoids induce peroxisome proliferator-activated receptor-coactivator (PGC)-1alpha gene transcription: An autoregulatory loop controls PGC-1alpha expression in adipocytes via peroxisome proliferator-activated receptor-gamma coactivation. Endocrinology.

[8] Schaum N., Lehallier B., Hahn O., Pálovics R., Hosseinzadeh S., Lee S.E., Sit R., Lee D.P., Losada P.M., Zardeneta M.E. (2020). Ageing hallmarks exhibit organ-specific temporal signatures. Nature.

[9] Yu Q., Xiao H., Jedrychowski M.P., Schweppe D.K., Navarrete-Perea J., Knott J., Rogers J., Chouchani E.T., Gygi S.P. (2020). Sample multiplexing for targeted pathway proteomics in aging mice. Proc. Natl. Acad. Sci. USA.

[10] Grimsrud P.A., Picklo M.J., Griffin T.J., Bernlohr D.A. (2007). Carbonylation of Adipose Proteins in Obesity and Insulin Resistance. Mol. Cell. Proteom..

[11] Hauck A.K., Olson D.H., Burrill J.S., Bernlohr D.A. (2017). Adipose Carbonylation and Mitochondrial Dysfunction.

[12] Wong C.-M., Marcocci L., Das D., Wang X., Luo H., Zungu-Edmondson M., Suzuki Y.J. (2013). Mechanism of protein decarbonylation. Free Radic. Biol. Med..

[13] Frohnert B.I., Sinaiko A.R., Serrot F.J., Foncea R.E., Moran A., Ikramuddin S., Choudry U., Bernlohr D.A. (2011). Increased Adipose Protein Carbonylation in Human Obesity. Obesity.

[14] Curtis J.M., Hahn W.S., Stone M.D., Inda J.J., Droullard D.J., Kuzmicic J.P., Donoghue M.A., Long E.K., Armien A.G., Lavandero S. (2012). Protein Carbonylation and Adipocyte Mitochondrial Function. J. Biol. Chem..

[15] Hauck A.K., Zhou T., Hahn W., Petegrosso R., Kuang R., Chen Y., Bernlohr D.A. (2018). Obesity-induced protein carbonylation in murine adipose tissue regulates the DNA-binding domain of nuclear zinc finger proteins. J. Biol. Chem..

[16] Gregoretti I., Gregoretti I.V., Lee Y.-M., Goodson H.V. (2004). Molecular Evolution of the Histone Deacetylase Family: Functional Implications of Phylogenetic Analysis. J. Mol. Biol..

[17] Kouzarides T. (2007). Chromatin Modifications and Their Function. Cell.

[18] Student A.K., Hsu R.Y., Lane M.D. (1980). Induction of fatty acid synthetase synthesis in differentiating 3T3-L1 preadipocytes. J. Biol. Chem..

[19] Shechter D., Dormann H.L., Allis C.D., Hake S.B. (2007). Extraction, purification and analysis of histones. Nat. Protoc..

[20] Rappsilber J., Mann M., Ishihama Y. (2007). Protocol for micro-purification, enrichment, pre-fractionation and storage of peptides for proteomics using StageTips. Nat. Protoc..

[21] Cox J., Mann M. (2008). MaxQuant enables high peptide identification rates, individualized p.p.b.-range mass accuracies and proteome-wide protein quantification. Nat. Biotechnol..

[22] Curtis J.M., Grimsrud P.A., Wright W.S., Xu X., Foncea R.E., Graham D.W., Brestoff J.R., Wiczer B.M., Ilkayeva O., Cianflone K. (2010). Downregulation of adipose glutathione S-transferase A4 leads to increased protein carbonylation, oxidative stress, and mitochondrial dysfunction. Diabetes.

[23] Liochev S.I. (2013). Reactive oxygen species and the free radical theory of aging. Free Radic. Biol. Med..

[24] Zheng J., Mutcherson R., Helfand S.L. (2005). Calorie restriction delays lipid oxidative damage in Drosophila melanogaster. Aging Cell.

[25] Zimniak P. (2011). Relationship of electrophilic stress to aging. Free Radic. Biol. Med..

[26] Ayyadevara S., Dandapat A., Singh S.P., Siegel E.R., Shmookler Reis R.J., Zimniak L., Zimniak P. (2007). Life span and stress resistance of Caenorhabditis elegans are differentially affected by glutathione transferases metabolizing 4-hydroxynon-2-enal. Mech. Ageing Dev..

[27] Austad S.N., Fischer K.E. (2016). Sex Differences in Lifespan. Cell Metab..

[28] Galligan J.J., Rose K.L., Beavers W.N., Hill S., Tallman K.A., Tansey W.P., Marnett L.J. (2014). Stable Histone Adduction by 4-Oxo-2-nonenal: A Potential Link between Oxidative Stress and Epigenetics. J. Am. Chem. Soc..

[29] Saltiel A.R., Olefsky J.M. (2017). Inflammatory mechanisms linking obesity and metabolic disease. J. Clin. Investig..

[30] James A.M., Collins Y., Logan A., Murphy M.P. (2012). Mitochondrial oxidative stress and the metabolic syndrome. Trends Endocrinol. Metab..

[31] Harman D. (1956). Aging: A theory based on free radical and radiation chemistry. J. Gerontol..

[32] Le Lay S., Simard G., Martinez M.C., Andriantsitohaina R. (2014). Oxidative Stress and Metabolic Pathologies: From an Adipocentric Point of View. Oxidative Med. Cell. Longev..

[33] Roberts C.K., Sindhu K.K. (2009). Oxidative stress and metabolic syndrome. Life Sci..

[34] Ostan R., Monti D., Gueresi P., Bussolotto M., Franceschi C., Baggio G. (2016). Gender, aging and longevity in humans: An update of an intriguing/neglected scenario paving the way to a gender-specific medicine. Clin. Sci..

[35] Viña J., Borrás C., Gambini J., Sastre J., Pallardó F.V. (2005). Why females live longer than males? Importance of the upregulation of longevity-associated genes by oestrogenic compounds. FEBS Lett..

[36] Huang H., Sabari B.R., Garcia B.A., Allis C.D., Zhao Y. (2014). SnapShot: Histone Modifications. Cell.

[37] Lu C., Thompson C.B. (2012). Metabolic Regulation of Epigenetics. Cell Metab..

[38] Zentner G.E., Henikoff S. (2013). Regulation of nucleosome dynamics by histone modifications. Nat. Struct. Mol. Biol..

[39] Fan J., Krautkramer K.A., Feldman J.L., Denu J.M. (2014). Metabolic Regulation of Histone Post-Translational Modifications. ACS Chem. Biol..

[40] Sims R.J., Nishioka K., Reinberg D. (2003). Histone lysine methylation: A signature for chromatin function. Trends Genet..

[41] Pan G., Tian S., Nie J., Yang C., Ruotti V., Wei H., Jonsdottir G.A., Stewart R., Thomson J.A. (2007). Whole-Genome Analysis of Histone H3 Lysine 4 and Lysine 27 Methylation in Human Embryonic Stem Cells. Cell Stem Cell.

[42] Zhao X.D., Han X., Chew J.L., Liu J., Chiu K.P., Choo A., Orlov Y.L., Sung W.-K., Shahab A., Kuznetsov V.A. (2007). Whole-Genome Mapping of Histone H3 Lys4 and 27 Trimethylations Reveals Distinct Genomic Compartments in Human Embryonic Stem Cells. Cell Stem Cell.

[43] Black J.C., Whetstine J.R. (2014). Chromatin landscape. Epigenetics.

[44] Gates L.A., Foulds C.E., O’Malley B.W. (2017). Histone Marks in the “Driver”s Seat’: Functional Roles in Steering the Transcription Cycle. Trends Biochem. Sci..

