# Supplementary figures and images for: Histone Carbonylation Is a Redox-Regulated Epigenomic Mark That Accumulates with Obesity and Aging
Source: Antioxidants (Basel). 2020 Dec 1;9(12):1210. doi: 10.3390/antiox9121210 (PMC7761391; doi:10.3390/antiox9121210)

# Supplemental Figure 1

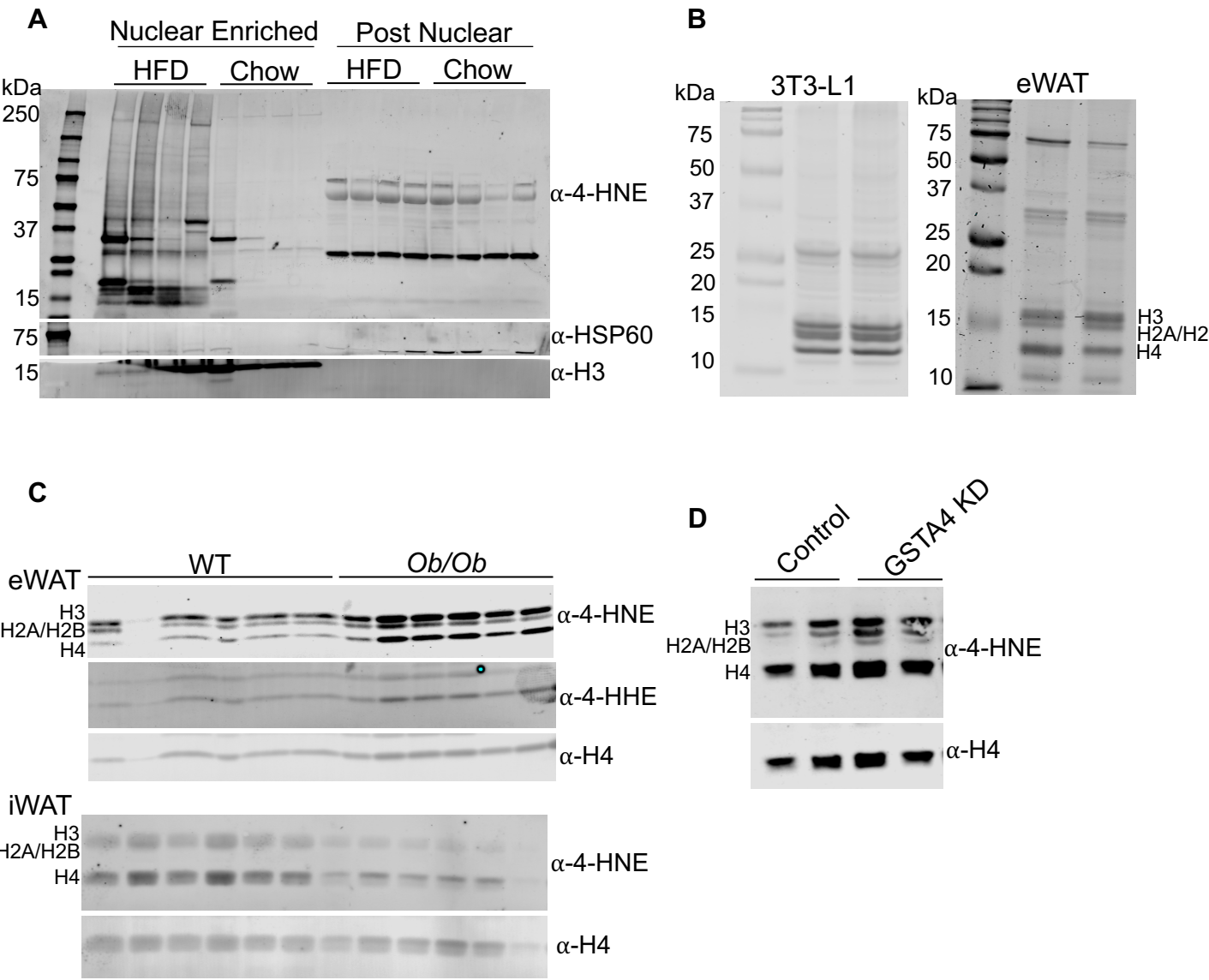

Supplement: Supplementary file 1 [file antioxidants-09-01210-s001.pdf]
